# Nepalese dental hygiene and dental students’ career choice motivation and plans after graduation: a descriptive cross-sectional comparison

**DOI:** 10.1186/s12909-015-0500-5

**Published:** 2015-12-11

**Authors:** Ron J. M. Knevel, Mark G. Gussy, Jane Farmer, Leila Karimi

**Affiliations:** 1 La Trobe University, School of Dentistry and Oral Health, PO Box 199, Bendigo, VIC 3552 Australia; 2 La Trobe University, Faculty of Health Sciences, PO Box 199, Bendigo, VIC 3552 Australia; 3 La Trobe University, Faculty of Health Sciences, Bundoora, VIC 3086 Australia

**Keywords:** Dental education, Dental hygienist education, Career motivation, Perception, Career expectation, Study motivation, Nepal

## Abstract

**Background:**

This is the first study of its kind to provide data regarding the self-reported career choice motivation and intentions after graduation of dental and dental hygiene students in Nepal. The findings of this study can be used to inform future oral health workforce planning in Nepal.

**Methods:**

A cross-sectional survey of dentistry and dental hygiene students attending a large accredited dental college in Kathmandu, Nepal. Quantitative data were analysed using IBM® SPSS® 22. The respondents were given the opportunity to provide clarifying comments to some of the questions.

**Results:**

Two hundred questionnaires were distributed, and 171 students completed the anonymous survey (response rate 86 %). Working in health care and serving the community were the most important initial motives for career choice, with significantly more dentistry students selecting their degree course because of the possibility to work flexible working hours (*p* < .001) compared to dental hygiene students. A majority of the students expressed concern about finding a suitable job (58 %) after graduation. Almost a quarter (23 %) reported intent to seek a job immediately after graduation, while 46 % plan further studies. Dentistry students were more likely to report planning further studies (*p* = .007) compared to the dental hygiene students. Dental hygiene students express a higher interest in going abroad (*p* = .011) following graduation. Only 10 % of all students plan to live or work in rural areas after study. Most common preferred locations to live after graduation are urban (33 %) or abroad (38 %). Data suggest a preference to combine working in a hospital with working in their own practice (44 %) while interest in solely working in their own practice is low (<2 %).

**Conclusion:**

Many students, though enthusiastic about their profession and expressing the ambition to serve the community, fear unemployment or envision better chances abroad. Most of the students in this study expressed a preference to live in an urban area after graduation. Findings indicate that strong measures are required to incentivise students to consider rural work.

## Background

Many factors may influence choice of a career in dentistry or dental hygiene. Factors may vary between countries and change over time [1]. This is the first study of its kind to provide data regarding the self-reported career choice motivation and intentions after graduation of dental and dental hygiene students in Nepal. This study was undertaken to investigate factors that drive Nepalese students’ choices to study dentistry or dental hygiene and to examine their career intentions after graduation. Gaining insight about the motives and future plans of (potential) students and collecting data about the possible retention of recent graduates is valuable for oral health workforce planning models. Students’ motivation to become a dental hygienist in developed or developing countries is under-researched compared to student’s motivation to become dentists. Understanding influences could assist in improving selection processes for recruitment of students for both courses. It could allow selection of those students who after graduation, most likely will contribute to the provision of dental health services to the community [2, 3]. Understanding the factors related to retention of qualified providers and their career plans can contribute to the development of strategies to address oral health workforce shortages in remote or rural areas or other underserved areas [4] and can have consequences for the development of health care systems [1]. The findings of this study can be used to inform future oral health workforce planning in Nepal.

Lack of data and policy to define the structure of the labour force and population health needs hinders the planning and successful introduction of oral health services in Nepal. To determine the number of oral health care professionals required, the oral health status of the population and barriers to care should be considered. The first and only Nepalese oral health survey was conducted in 2004 [5]. Several small research projects to assess the oral health needs of the population [6–13] have also been undertaken but findings cannot be extrapolated to nationwide community needs. Recent expansion of the number of dental hygiene and dental colleges has led to an increase of the number of dental hygienists and dentists. This gives the impression that Nepal is trying to build a dental workforce to improve access to dental care to meet the population needs, although the government has not implemented an oral health workforce-planning model. The public health sector in Nepal can’t fulfil the demand for care, with the private sector offers treatment at high costs. Most health care financing relies on household out-of-pocket payments [14]. Limited free public services are available, however not every district is able to offer dental care. The earthquake in Nepal in April 2015 might further affect the priority given to dental policies.

Dental education in Nepal was established in 1997, with the Peoples Dental College, Kathmandu, starting a Bachelor of Dental Surgery (BDS) program [15]. By 2008 five dental colleges had been established [15]. There are now 12 dental colleges (2014), each with an average intake of approximately 40 students [5]. According to the website of the Nepal Medical Council (NMC), three independent Dental Colleges were recognised. The other Dental Colleges are integrated in Nepal’s Medical Schools [16]. The Bachelor of Dental Surgery (BDS) is a 5 ½ undergraduate degree program, including a compulsory internship of 1 year. The training of dental hygienists was introduced to Nepal in 2000 [17]. In 2014 there were six dental hygiene colleges. Reliable numbers of dentists or dental hygienists working in Nepal are difficult to obtain. It is compulsory for the NMC to report an Annual Register; however since 2001 the NMC has not been able to do this [18]. Interviews with key informants in this area confirm that Nepal is struggling to keep track of their graduates. Political instability and lack of resources play an important role in this lack of infrastructure, a similar picture to developing economies in Africa [19]. This problem is not unique to Nepal. In general there seems to be a lack of information about indicators relevant to oral health between FDI World Dental Organisation (FDI) participating countries regardless of the level of economic development of the country [20]. Figures related to distribution of oral health workforce are lacking for many parts of the world [21]. Data for 2000 show that Nepal had 100 registered dentists, with a dentist per population ratio of 1:200.000 [22]. In 2008 the number of dentists registered with the medical council was reported between 624 and 705; 386 (421) females and 238 (284) males [7, 18]. According to WHO Oral Health Country/Area Profile Programme (CAPP), the ratio of dentist per inhabitant in Nepal is 1:47306 [23]. The Nepal Dental Association (NDA) reported that the number of dentists registered with the NMC is approximately 1550 (obtained via personal communication in July 2014). The number of registered dentists does not reflect the actual number of dentists working in Nepal. They might not be registered with the NDA or have emigrated [5]. In the World Health Statistics report from the WHO (2012 and 2013) the number of dentists in Nepal is not reported [24, 25].

Current estimates might also not reflect the total number of practicing dental hygienists. Since the introduction of dental hygiene programmes in 2000, approximately 900 dental hygienists have graduated. The Nepal Council for Technical and Vocational Education and Training (CTEVT) is responsible for accrediting dental hygiene programmes. Until 2014, students interested in becoming a dental hygienist could select to attend either a 2-year diploma course (Diploma Dental Hygiene, with a lower entry level) or a 3-year certificate course (Certificate of Dental Science, CDS). The 2-year course led to a Technical School Leaving Certificate in Dental Hygiene. The 2-year course allowed students with lower results in the School Leaving Certificate (SLC) examinations to enter the vocational dental hygiene course [26]. In many European countries or countries in the European Economic Area there has been a trend to prolong the length of the training of dental hygienists and to increase the tasks they can perform [27]. Nepal followed this trend in 2005 with the introduction of a 3-year course and the addition of atraumatic restorative treatment to the traditional scope of dental hygiene practice. The course results in a Certificate of Dental Science and enables students to continue to study any Bachelor program in Nepal providing the equivalent to college or university entry qualifications [26]. In 2014 the 2-year diploma course was discontinued.

## Methods

A group of dentistry (DEN) and dental hygiene (DH) students at one accredited Dental and Dental Hygiene College in Kathmandu was asked to participate in this study. Students were informed of the aim of the study and were provided with a participant information sheet and a consent form. Participation was voluntary and the survey-instrument was anonymous. The questionnaire was distributed to students in September 2013. Ethical approval for this study was obtained from the La Trobe University Human Ethics Committee (UHEC, 12-119a). Access to the students was gained to distribute a self-administered structured and pretested questionnaire, in English, during a lecture or a pre-clinical class. Comprehension of English was assumed given that most parts of the education program are conducted in English. The questionnaire was pretested in October 2012 at three different colleges in Nepal with 100 participants, after which small changes to the questionnaire were made, mostly relating to ambiguous language compromising understanding and allowing participants the opportunity to provide individual comments. The questionnaire was based on surveys used in similar research in other developed and developing countries. The students submitted their completed or incomplete survey in a closed box ensuring coercion-free participation. Neither the teacher nor researcher was able to identify if a student completed the form or not when the survey was returned.

The quantitative data were analysed using IBM® SPSS® 22. Descriptive statistics were used to report percentages of respondents on selected variables. Chi-square statistics or Fischer’s exact test were computed to determine differences between the groups of students. The additional individual comments to some of the close captured questions were used with the intention to provide context and understanding of the quantitative results of the survey or to reveal issues that might need further exploration [28, 29].

## Results

### Response rate and demographics

At the time of the study 478 students were enrolled in the dental college: 97 males (20 %) and 381 females (80 %). Two hundred surveys were distributed (to students present at specific lectures or practical sessions) and 171 completed questionnaires were returned resulting in a participation rate of 86 %. This means that 35 % of the total student population from this dental college participated in the study. Responses were received from 85 dental hygiene (DH) students (50 %) and 86 dentistry (DEN) students (50 %). Of respondents, 14 % were male and 86 % female. Chi-square analysis did not reflect a statistically significant difference between the gender balance of the sample population and the total number of students in this dental college. Table 1 summarises demographic details.

**Table 1:** Participation rates and demographics

	DH/CDS students	DEN students	Total students	
*N* = 171^a^				
Number of students per year	1^st^ 48 (56 %)	1^st^ 3 (4 %)		
	2^nd^ 23 (27 %)	2^nd^ 45 (52 %)		
	3^rd^ 14 (17 %)	3^rd^ 11 (12 %)		
		4^th^ 2 (2 %)		
		5^th^ + 24 (30 %)		
Response rate - *N* = 200	85	86	171 (86 %)	
Gender	♂ 16 (19 %)	♂ 8 (9 %)	♂ 24 (14 %)	
	♀ 69 (81 %)	♀ 77 (90 %)	♀ 147 (86 %)	
Age (mean, years)	18.9	20.7	19.8	
Caste/ethnicity:^b^				
Didn’t answer	23 (26 %)	26 (30 %)	49 (29 %)	
Newar (5.4 %)	14 (16 %)	21 (24 %)	35 (21 %)	
Brahmin (12.5 %)	8 (9 %)	15 (17 %)	23 (13 %)	
Chhetri (15.5 %)	6 (7 %)	14 (16 %)	20 (12 %)	
Other	9 (11 %)	4 (5 %)	13 (7 %)	
Gurung	9 (11 %)	1 (1 %)	10 (6 %)	
Magar (7 %)	6 (7 %)	3 (4 %)	9 (5 %)	
Mongolian	6 (7 %)	1 (1 %)	7 (4 %)	
Tamang (5.5 %)	4 (5 %)	0 (0 %)	4 (2 %)	
Tharu (6.6 %)	1 (1 %)	1 (1 %)	2 (1 %)	
	DH/CDS Students	DEN Students		*P*
Highest level education parents:				< .001^c^
Bachelor or higher	12 (14 %)	51 (60 %)		
Self-reported financial problems	44 (26 %)	34 (20 %)		0.27^c^
Borrowing money for study	33 (19 %)	32 (19 %)		0.85^c^
Diploma or less	73 (86 %)	30 (35 %)		

^a^Valid cases range from 163 to 171 (due to missing cases i.e. unanswered constructs)^b^2001 census

^c^Fisher's exact test

The mean age of the participants was 19.8 years. The mean age of the dental hygiene students was slightly lower (mean 18.9, range 15-29) compared to the mean age of the dentistry students (mean 20.7, range 18-27). First year dental hygiene students and second year dentistry students formed the largest proportions of the participants within this study; however each year level in both courses was represented to some degree. A difference in the level of parental education between groups was observed. Significantly more (Exact Fisher Test, *p* = .001) dentistry students (*n* = 51) reported that the level of the education of their parents was at least of bachelor or university level compared to dental hygiene students (*n* = 12). Most of the participants in this study (46 %) reported to belong to the Brahmin, Chettri or Newar caste/ethnicity.

In almost all cases parents financed students’ study. Only four per cent report being supported by an NGO, the government or other source. To support their studies 38 % of the students reported borrowing money, and 46 % of the students reported experiencing financial problems. These were not further investigated. There is no statistically significant difference between the dental hygiene or dentistry students regarding these *self-reported* financial problems or the fact that money is borrowed to support study.

### Reason for selection study and reported plans after graduation

The primary reasons for selecting dentistry or dental hygiene was a desire to work in health care (40 %) and to serve the community (31 %). Good reward (16 %) and increased chances to go abroad (15 %) were also important (see Table 2) as illustrated by the following example from the additional comments:

**Table 2:** Study motivation, intentions and employment expectation after graduation

N = 171^a^	DH/CDS students	DEN students	Total students	*P*
Study motivation:				<.001^c^
Want to work in healthcare	34 (20 %)	34 (20 %)	68 (39.8 %)	
Best way of serving community	28 (16.5 %)	24 (14.1 %)	53 (31 %)	
Reward	10 (5.9 %)	17 (10 %)	27 (15.8 %)	
Increase chances of going abroad	17 (10 %)	9 (5.3 %)	26 (15.2 %)	
Job security	10 (5.9 %)	11 (6.5 %)	21 (12.3 %)	
Flexible working hours	3 (1.7 %)	17 (10 %)	20 (11.7 %)	
Status/prestige	9 (5.3 %)	9 (5.3 %)	18 (10.5 %)	
Further study	12 (7 %)	6 (3.5 %)	18 (10.5 %)	
Peer/family pressure	1 (0.6 %)	4 (2.4 %)	5 (2.9 %)	
Don’t know	1 (0.6 %)	2 (1.2 %)	3 (1.8 %)	
Plan to live after completion course:				.327^b^
Abroad	33 (38.8 %)	29 (34.1 %)	62 (36.3 %)	
Kathmandu Valley	18 (20 %)	14 (16.5 %)	32 (18.7 %)	
Undecided	14 (16.5 %)	15 (17.6 %)	29 (17 %)	
Home town, urban	7 (8.2 %)	16 (18.8 %)	23 (13.5 %)	
Home town, rural	10 (11.8 %)	7 (8.2 %)	17 (9.9 %)	
Not answered	4 (4.7 %)	3 (4.7 %)	8 (4.7 %)	
Plan after completion course:				.007^b^
Further study	26 (30.6 %)	43 (50.6 %)	69 (40.4 %)	
Seeking job	16 (18.8 %)	18 (21.2 %)	35 (20.5 %)	
Going abroad	25 (29.4 %)	9 (10.6 %)	34 (19.9 %)	
Undecided or other	18 (21.2 %)	13 (15.3 %)	31 (18.1 %)	
Not answered	0	2 (2.4 %)	2 (1.2 %)	
Intention to work after graduation:				.011^c^
Both own practice and existing dental hospital	31 (36.5 %)	43 (50.6 %)	75 (43.9 %)	
Existing dental hospital	16 (18.8 %)	22 (25.9 %)	38 (22.2 %)	
Abroad	22 (25.9 %)	8 (9.4 %)	30 (17.5 %)	
Undecided or other	7 (4.4 %)	6 (7.1 %)	17 (9.9 %)	
Not answered	1 (1.2 %)	3 (3.5 %)	8 (4.7 %)	
Own practice	3 (3.5 %)	0 (0 %)	3 (1.8 %)	
Worried about finding a job:				.430^b^
Yes	49 (57.7 %)	50 (58.8 %)	99 (57.9 %)	
No	17 (20 %)	23 (27.1 %)	41 (24 %)	
Don’t know Or not answered	19 (22.3 %)	12 (14.1 %)	27 (15.8 %)	

Percentages may not total 100 % because of rounding or multiple answers

^a^Valid cases range from 163 to 171 (due to missing cases i.e. unanswered constructs)

^b^Chi-Square

^c^Fisher’s exact test

*“Best way of serving community and it also offers good status. We will develop our own identity. If possible we can go abroad for further education.” (DH70)*

Flexible working hours (12 %), enabling further study (11 %) as well as status and peer or family pressure seem to play a less important role.

Analysis of study motivation for each group found that the only significant difference between dentistry students and dental hygiene students was related to the opportunity to work flexible working hours after graduation. Significantly more dentistry students (10 %, *n* = 17) reported that flexible working hours was a reason to study dentistry compared to the dental hygiene students (2 %, *n* = 3), *χ*^2^ (1, *N* = 170) = 11.107, *p* < .001.

Most students reported that the selection of study area was mainly their own decision (77 %). However for 61 % parents had ‘some’ or ‘much’ influence on study selection. Siblings influenced study for 44 % of respondents. There were no significant differences between student groups.

Following course completion, 36 % planned to live abroad, while 31 % planned to live in Kathmandu Valley or other urban Nepal. Only 10 % plan to live and work in rural areas, with 17 % undecided. After graduation, only one in five (21 %) intend seeking a job immediately, while 40 % are interested in further studies. With regard to the relationship between study area and interest in going abroad, dental hygiene students (29 %, *n* = 25) reported significantly higher interest in planning to go abroad (*n* = 149), *χ*^2^ (1, *N* = 171) = 12.015, *p* = .007, while dentistry students (51 %, *n* = 43) reported significantly higher interest in further studies.

Asked about choice of work setting after graduation, 44 % overall prefer to combine working in their own practice with working in a dental hospital. None of the dentistry students reported an ambition to solely work in their own practice. Significantly more dental hygiene students (26 %, *n* = 22) reported plans to finding a job in another country compared to dentistry students (9 %, *n* = 8), *χ*^2^ (1, *N* =158) = 12.161, *p* = .011. The additional comments of dental hygiene students expand on these findings and provide insights into possible reasons for this. One of the most cited additional comments (cited by 22 students) from dental hygiene students revealed that the lack of a Code of Ethics^1^ for Dental Hygienists with a Technical School Leaving Certificate in Dental Hygiene (2-year course) is an important issue:

*“The profession is not so good in Nepal… In Nepal there is no code of ethics for the dental hygienist. I can only hope it will be good in the future. I will try my best and hope my dreams come true.” (DH20)*

*“It is an important profession, but in Nepal dental hygienists don’t have a good identity. It is not regulated. Dental hygienists are fighting for their identity. Till today we don’t have a code of ethics.” (DH19)*

*“Quite often dental hygienists go abroad to get a better education and a job, even though they are very talented in the field of dental hygiene. The scope of practice of the dental hygienist is not so good in Nepal and getting a job is difficult.” (DH5)*

*“In context of Nepal the position of the dental hygienist is poor… There are no government posts for dental hygienists.” (DH1)*

Of responding students, 58 % report worry about finding a job, with no significant difference between DEN and dental hygiene students; 22 dentistry students provided additional comments to this question and 95 % of these comments were related to self-perceived increased competition.

*“There are more dentists produced each year and there is not a good job offer for dentists.” (DEN97)*

*“Dental manpower is getting more, whereas job vacancy is low, and the salary is also not suitable” (DEN143)*

## Discussion

Our finding that parental level of education of dentistry students was significantly higher, than that of dental hygiene students’ parents may indicate that dentistry students tend to be from a higher educated family background. The level of education of the parents seems the major determinant in the selection of a higher level of study and may contribute to the selection of university-level study over certificate or diploma level training. The majority of students (64 %) reported that the choice to study dentistry or dental hygiene was their own, while one third (33 %) indicated that parents had a high level of influence on study decisions. Although it was expected that Nepalese parents have a high influence on their children study decisions, only 2.5 % of the dentistry students thought peer/family pressure influenced study choice, suggesting that decisions to study dentistry or dental hygiene tend to be autonomous. These findings are similar to the findings of a recent study amongst medical students in Nepal where family influence on career/study selection seems of small impact [30].

The results show that a majority of the dentistry students are from families with greater educational achievement, which can be a proxy for social position. As with other studies [31, 32] students enrolled in higher education tend to be from families of higher social position, which is evidenced by the fact that a majority of the students (46 %) in this study reported to be a part of the Brahmin, Chhetri or Newar caste. According to Central Bank of Nepal (2013) Newar, Brahmin and Chhetri have the highest monthly earning in Rupees per month compared to other ethnic groups or caste and their level of education is higher [33]. In our study only a minority (four per cent) reported financial support from the government or other sources (NGO, scholarship).

In other international studies, choosing dentistry is often related to the social status and lifestyle of the profession, as much as the desire to serve others and work in health [1, 2, 4, 31, 34, 35]. A recent study of Australian Bachelor of Oral Health (BOH) students showed that caring and helping other people was the primary motivation for study selection [36]. Findings here reveal that working in health care and interest to serve the community were most commonly cited as a reason to study dentistry or dental hygiene. Opportunity to work abroad or enabling further studies was cited by 15 %. Good reward and status seem to play a slightly less important role.

In 2008, 60 % of Nepalese registered dentists were female [7, 18]. At the time of our survey, 80 % of the enrolled students at this dental college were female, which is consistent with global trends of greater numbers of female students in dentistry training [31, 35, 37–43]. The specific characteristics of the dentistry profession (higher degree of flexibility, possible more autonomy compared to dental hygienists and balance between personal and professional life) can be important in selecting a degree [42, 43]. This might explain why significantly more dentistry students (20 %) included flexible work hours as part of their study motivation compared to dental hygiene students (4 %). Working hours of female dentists tend to decrease when they have children and female dentists are more likely to take a career break [38]. Some researchers speculate that men and women practice differently, with fewer women interested in owning their own practice and more likely to prefer working in the urban areas [44]. These specific lifestyle choices by female dentists might impact oral health workforce supply.

Interest in going abroad, for further studies or job opportunities, is high amongst the participants in this study. In general many Nepalese nationals travel abroad for higher studies due to a perceived lack of quality of domestic institutions, shortage of places at the best institutions, political instability and politicisation of universities [45]. These push and pull factors for migration are influenced by socio-political and educational factors [19] and this possible brain drain from migration of dental hygiene and dentistry graduates (or “loss of intellectual capital”) has potential to compromise the ability to build a successful oral health workforce in Nepal and hinder the provision of adequate (preventative) oral health services. The increase of educational institutions for health training in Nepal has resulted in a larger number of trained health professionals; however the number of jobs in the sector has not increased, leading to unemployment and possible loss of interest to study for this type of qualification [46]. One strategy for graduates who do not immediately secure employment is to pursue further education. Our study shows that 40 % of students plan further studies following graduation. Other relevant factors cited by the participants are the lack of governmental positions and perceived increased competition for jobs due to the expansion of the number of education programmes; both contributing to the reported fear of finding a suitable job by 58 % of the students.

It is noteworthy that more dental hygiene students in this study are interested in going abroad when compared to dentistry students. In most countries the role of dental technicians and dental nurses has been well defined but not so for the dental hygienist [27]. Currently the lack of a Code of Ethics for the dental hygiene graduates in Nepal from the 2-year course and the incorrect Code of Ethics for the CDS graduates (3-year course), are a major source of professional frustration, dissatisfaction and potential conflict between professional organisations. Newer (para-professional) health workers often are restricted in scope of practice and encounter resistance from the more established professional groups [47]. The belief of trainees that they are not valued within the health care system inevitably leads to demotivation [48]. The lack of a Code of Ethics for graduates from the 2-year course potentially affects their expectations for a suitable career. The different social status of the relatively new dental hygiene profession in Nepal might have influenced the dental hygiene student’s expectations in this study.

The majority of the dentistry students expressed interest in working in existing dental hospitals in combination with independent practice. Dentistry students in this study seem to show no interest in solely working in their own practice. Commonly cited reasons provided by dentistry students in additional comments demonstrate that they feel that opening a private practice is hard and the lack of awareness of Nepalese people about oral health might prevent earning a proper income. Working in an established dental hospital could raise one’s professional status and at the same time offer the opportunity to learn from experienced dentists and improve professional skills. The intention from this group of dentistry students seems to differ from the reported trend that dentists in developing countries prefer to work in private practice [21]. According to a recent survey by the FDI geographical mal-distribution of dentists is a problem regardless of the economic development of a country and migration to larger cities is an international trend [21]. A majority of this study’s students preferred to work in urban areas, a finding that aligns with other studies [4, 21, 31, 35, 36, 49, 50] with only 10 % intending to work in rural areas after graduation. This will not benefit the 80 % of Nepalese people that live in rural areas [51]. Strategies can be developed to try to select these students who most likely will work in rural settings. For these strategies to succeed it is imperative that the government is successful in creating positions in rural areas. The growth of mostly private dental schools has increased inequalities in access with greater access for urban residents [52], but continuing poor access in rural areas. Although the overall number of oral health professionals is increasing their distribution remains uneven, leading to worsening inequalities [52] consistent with the Inverse Care Law that says “the availability of good medical care tends to vary inversely with the need for it in the population served” [53]. Mal-distribution will continue to undermine the provision of equitable rural health care services [48]. The Ministry of Health posted dental surgeons studying in governmental colleges to district hospitals in 2007. Not all the posts were filled: poor infrastructure, lack of incentives, lack of career opportunities, political instability and poor infrastructure contributed to problems with recruiting dental personnel to work in rural areas [7]. The findings reflect a vicious cycle, in which government or rural job opportunities hardly exist and the working conditions are unattractive. This leads to a growing interest in working in metropolitan areas, which in turn can even further reduce the interest of the government in establishing posts in rural areas. Developing career and incentive systems, with better social and technical support for health workers has been suggested [48] to attract health workers to rural areas. If shortages persist the high workloads in the rural areas could lead to poor quality of care and low morale of the workers due to the inappropriate working environment and overall low job satisfaction [48, 54]. Recently created government positions in several districts attracted many applicants. In the proposal for the new Nepalese National Oral Health Policy (2014) several position are included for trained oral health professionals (dentists, dental hygienists or skilled dental aides). Whether the policy will be implemented remains uncertain.

## Limitations

The study had some limitations. Data were obtained using a self-administered questionnaire with students from different year levels at one Nepalese dental college using a convenience sample. Findings may not be generalisable nationally as students sampled were enrolled in a single institute. Further research across different educational locations would be beneficial. This study was a cross section of students in different years of their study. No differentiation was made between students and year levels. Further investigation should compare motivational factors and perception differences between different years of study and perhaps even gender to investigate a difference between the intentions after graduation of female and male students. However this study provides insights into oral health workforce issues in a developing country and shows differences between two emerging oral health professions in Nepal. It would be useful to extend the study to other educational sites in Nepal and to follow cohorts of students over time to monitor what happens to the students when they transition into the workforce.

## Conclusion

This study, though limited and including a single setting, provides important information because it is the first of its kind to be written up in relation to Nepal. It provides useful indications why Nepalese students want to enter the profession, but also provides insight in their concerns. This study shows that the primary motivating factor for choosing dentistry or dental hygiene for Nepalese students is the ambition to work in health care and to serve the community. The students reported a perception that the rapid growth in the number of dental and dental hygiene programmes in Nepal could limit their chance for a reasonable income. A majority of the students fear not finding a job after graduation. This could encourage students to pursue further studies or emigrate to acquire a higher standard of living. It is concerning that so many students, though enthusiastic about their profession and expressing the ambition to serve the community, fear unemployment or envision better chances abroad. Governments, professional organisations and decision makers should take steps to counter these movements out of the profession.

Prospects look bleak for needy rural populations. Most of the students in this study expressed a preference to live in an urban area after graduation. Findings indicate that strong measures are required to incentivise students to consider rural work. The motivations from students should be taken into account if governments want to retain the students within Nepal. They need to consider how to build these factors into job opportunities. Further study of more Nepalese colleges is planned to ascertain if the indications from this study are more generalisable. The collected data can support the government or leadership to take some very concrete steps to ensure that statistics on the oral health workforce are updated annually and policies are formulated about the required number and outputs of dental colleges.

## Footnotes

1 The “Code of Ethics” is published by the Nepal Health Professional Council and describes the minimum qualification and training objectives for the CDS graduate. It describes the scope of practice. It is expected that CDS graduates provide quality dental care services to the population based on the specified qualifications only.
